# Intrauterine Growth Retardation Complicated by Biermer's Disease: An Observation in Togo

**DOI:** 10.1155/2019/4539675

**Published:** 2019-05-06

**Authors:** Essohana Padaro, Baguilane Douaguibe, Kossi Agbétiafa, Irénée Kueviakoe, Ahoefa Vovor

**Affiliations:** ^1^Hematology Department, Campus Teaching Hospital, University of Lome, Lome, Togo; ^2^Gynecology Department, Campus Teaching Hospital, University of Lome, Lome, Togo; ^3^Hematology Department, Tokoin Teaching Hospital, University of Lome, Lome, Togo

## Abstract

**Objective:**

To report the first case in Togo of Biermer's disease associated with intrauterine growth retardation (IUGR) in a 39-year-old pregnant woman.

**Observation:**

The patient with phenotype AA, born on 20/02/1978, G_2_P_0_ (a spontaneous abortion at 3 months), was referred to hematology on 17^th^ March 2017 for anemia at 26 weeks of amenorrhea (WA). She had received martial treatment with ferrous fumarate 66 milligrams daily. At 26 weeks, the uterine height was 16 centimeters, and there was good fetal vitality. During ultrasound, there was a harmonious development of the fetus, but it was lower than that for the gestational age at 10^th^ percentile based on fetal biometry, and anemia was at 65 g/l. She was then referred to hematology where she was found to have pancytopenia with macrocytic aregenerative anemia at 47 g/l (MCV at 109 fl), neutropenia at 1.02 g/l, and thrombocytopenia at 58 g/l. The myelogram found megaloblastosis at 53%, collapsed serum B12 vitamin at 61.7 pg/ml, normal serum folate at 9.9 ng/ml, increased serum homocysteine to 51.44 *μ*mol/l, and elevated LDH. The search for intrinsic anti-factor antibodies was positive. Digestive endoscopy noted fundal atrophy. The patient was given vitamin B12 injection; at the 7^th^ day, hemoglobin was observed at 94 g/l, then a normalization of the blood count after 3 weeks, and a good evolution of the pregnancy with delivery at 38 WA of a newborn, female, weighing 1960 g with 500 grams of placenta, with a size of 40 cm, and a cranial perimeter of 29 cm. The child had stunted weight growth (at 16 months; weight = 7 kg; height = 69 cm).

**Conclusion:**

Biermer's disease as a maternal cause of IUGR and infantile hypotonia is a reality in Togo. It requires management in patients and especially during pregnancy to avoid neurological complications in children born from mothers with this disease.

## 1. Introduction

Biermer's disease (BD) also called pernicious anemia is an autoimmune atrophic gastritis with dryness of Castle's intrinsic factor (IF) responsible for vitamin B12 deficiency. It is usually diagnosed in the presence of megaloblastic anemia, neurologic symptoms, or atrophic gastritis [1]. This entity accounts for more than 25% of the etiologies of vitamin B12 deficiency in adults and results from malabsorption caused by autoimmune gastritis [2]. It is manifested by anemic and/or digestive and/or neurological signs. The diagnosis is based on the demonstration of chronic atrophic gastritis, the positivity of anti-FI (anti-intrinsic factor) and/or anti-GPC (antigastric parietal cell) autoantibodies in the serum, and/or the decrease of selective vitamin B12 [1]. The association of Biermer's disease with autoimmune diseases such as type 1 diabetes (insulin-dependent), autoimmune thyroiditis (especially Hashimoto's), or vitiligo is common [3]. Through an increase of homocysteine induced by vitamin B12 deficiency, several studies have reported venous thrombosis during Biermer's disease [1, 4]. During pregnancy, the causes or factors associated with an increased risk of intrauterine growth retardation (IUGR) are extremely numerous and can be grouped into maternal, placental, and fetal anemia [5]. Maternal anemia, irrespective of its origin, is one of the causes of IUGR and neonatal hypotrophy. Anemia in pregnant women is detrimental to fetal growth and pregnancy outcomes [6]. Low birth weight and preterm delivery have been persistently linked to anemia in pregnancy [7]. Chronic maternal diseases include heart disease and chronic hypertension, SLE, diabetes with vascular complications, chronic nephropathies, proven thrombophilias, chronic respiratory diseases, and also chronic gastrointestinal and intestinal pathologies, including Biermer's disease [5].

In Togo, a recent study reported cases of Biermer's disease in its classic presentation [8]. In the literature, no reports of Biermer's disease associated with IUGR have already been reported in Togo. We report the first observation of Biermer's disease associated with IUGR in a 39-year-old pregnant woman.

## 2. Observation

The patient, born on 20/02/1978, was referred to hematology of the Campus Teaching Hospital by the gynecologist on 17^th^ March 2017 for an exploration of severe chronic anemia. She was pregnant and at 26 WA. She was a second gesture, nulliparous, and antecedent of spontaneous abortion at 3 months. She was seen in gynecology at 13 weeks of amenorrhea (WA). She had a regular cycle, the date of the last menstruation well known confirmed by the ultrasound of the first trimester. Physical examination revealed conjunctival paleness and moderate asthenia. The prenatal checkup blood count showed anemia at 7 g/l, the deep drop the parasitological examination of the stools were without abnormalities; and the electrophoresis of hemoglobin (Hb) showed a normal Hb AA. She was not an alcoholic; she had received martial treatment with ferrous fumarate 66 milligrams per day. At 26 WA, clinical examination showed the persistence of mucous pallor and asthenia, a weight of 57 kg, a height of 163 cm, a blood pressure (BP) of 120/60 mm·Hg in both arms, and negative proteinuria. Obstetrically, the uterine height was 16 cm, and there was good fetal vitality. During ultrasound, there was a harmonious development of the fetus, but it was lower than that for the gestational age at 10^th^ percentile based on fetal biometry. The amniotic fluid amount was normal. The uterine artery Doppler and the fetal brain Doppler were normal. The control hemogram had objectified anemia at 65 g/l. In view of etiological research of anemia, this patient was referred to hematology before the signs of IUGR complication.

The clinical examination in hematology had found the relatively compensated anemic syndrome. The general condition was good. There were no digestive or neurological signs. After hematological examination, pancytopenia with macrocytic aregenerative anemia 47 g/l (MCV at 109 fl) (reticulocytes at 23 g/l), leukopenia at 3.4 g/l, neutropenia at 1.02 g/l, and thrombocytopenia at 58 g/l were observed. The myelogram by sternal puncture found megaloblastosis at 53%. On a biochemical basis, the serum vitamin B12 assay showed a collapsed vitamin B12 at 61.7 pg/ml (208–963.5), normal serum folates at 9.9 ng/ml (7.2–15.4), increased serum homocysteine to 51.44 *μ*mol/l (3.36–20.44 *μ*mol/l), and elevated LDH (6044 IU/l) > 13 normal. Immunological marking of anti-intrinsic factors (IF) performed at the CERBA Laboratory in France was positive. Upper gastrointestinal endoscopy with biopsies noted fundal atrophy with erosive bulbitis. Anatomopathological examination showed chronic atrophic gastritis without *Helicobacter pylori*. The anti-IF antibody had not been researched on biopsy specimens. The diagnosis of BD had been posited with certainty. IUGR was secondary to this chronic anemia related to BD.

The patient was given vitamin B12 deep intramuscular injection (1000 *μ*g daily during 10 days and then a monthly injection for lifetime). Under this treatment, reticulocyte crisis was noted at the 7^th^ day (reticulocytes at 94.3 g/l) with a hemoglobin rate of 94 g/l and a gradual normalization of the blood count after 3 weeks. On the gyneco-obstetrical level, good evolution of the pregnancy was noted, which resulted in a delivery on 3^rd^ June 2017 by caesarean section at 38 WA of a newborn, female, of 1960 grams with 500 grams of placenta. The size of the newborn was 40 cm with a cranial perimeter of 29 cm. In October 2018, the child had a stunted weight loss because at 16 months, the child weighed only 7 kg for a height of 69 cm. However, she had good neurological development.

## 3. Discussion

Biermer's disease (formerly called pernicious anemia) is an autoimmune atrophic gastritis, predominantly fundic, responsible for vitamin B12 deficiency (cobalamin) by malabsorption of the latter [1]. Vitamin B12 is the cofactor of methylmalonyl mutase and methionine synthetase. Vitamin B12 is a ubiquitous coenzyme involved in a large number of intracellular enzyme reactions [9]. These are essentially the reactions that lead to the synthesis of DNA and the synthesis of methionine from homocysteine. Vitamin B12 deficiency is responsible for elevated homocysteine, methylmalonic acid, and propionylcarnitine [10]. B12 hypovitaminosis is common in adults and the elderly, with prevalence ranging from 15 to 40%, depending on the studies and the definition used [10]. It is often not diagnosed early because of poor clinical manifestations. However, its potentially serious hematological and neuropsychiatric complications require the diagnostic investigation of any patient with vitamin B12 deficiency, irrespective of the cause. Biermer's disease accounts for 25% of the etiologies of vitamin B12 deficiencies in adults according to the series [2]. The prevalence of Biermer's disease is 0.1% in the general population and 1.9% in subjects over 60 years of age [10]. Alteration of DNA synthesis results in blockage of maturation affecting mainly fast-multiplying cells and gives rise to hematological and mucocutaneous syndromes. Hematologically, DNA synthesis blockade and conserved synthesis of RNA are at the origin of cytoplasmic nucleotide asynchrony. Intramedullary abortion linked to inefficient hematopoiesis and megaloblastosis is responsible for clinicobiological images that can simulate true hemolytic anemia [11]. The patient has biological signs of hemolysis including an increase in LDH.

On the obstetrical side, recent studies have shown the effects of homocysteine on the fetus. Indeed, homocysteine results from the transmethylation of methionine. Its metabolism depends primarily on three enzymes and several vitamin cofactors. Genetic abnormality in these enzymes or deficiency of these vitamins leads to hyperhomocysteinemia (HHCh). HHCh is usually biologically defined by a fasting value >15 *μ*mol/l. HHCh is among the congenital hypercoagulable states and is a long-known vascular disease risk factor. The discovery that HHCh may also be responsible for several pregnancy complications has only recently been made [12]. Studies in this area are still scarce and report on limited numbers of patients. It nevertheless appears clear that HHCh is associated with the syndromes of repeated miscarriage, preeclampsia, placental abruption, thromboembolic events, neural tube defects, and perhaps fetal death in utero and intrauterine growth retardation [12].

Also, methyl donors (B12 and folates) regulate the monocarbon cycle, which plays a key role in epigenetic/epigenomic regulation by methylation [13]. Methyl donor deficiency produces intrauterine growth retardation and promotes developmental abnormalities, mainly of the central nervous system. In addition, high levels of homocysteine associated with such deficiency are a risk factor for various neurodegenerative pathologies. The authors studied the consequences of periconceptional and gestational deficiency on the embryonic brain development in Wistar rats [13]. Morphometric study showed retarded growth of deficient embryos that also affected the brain, with atrophy of structures such as the hippocampus, cortex, and subventricular zone. In addition, they demonstrated that methyl donor deficiency was associated with a posttranslational modification corresponding to an irreversible *N*-homocysteinylation of neuronal proteins, in particular, associated with the cytoskeleton. This modification induces the aggregation of proteins, a phenomenon involved in many neurodegenerative diseases. The combination of these different mechanisms sheds new light on the developmental defects and cognitive disorders associated with early methyl donor deficiency, highlighting the importance of “fetal programming” in the occurrence of certain neurological pathologies [1, 12]. The patient had an increase in homocysteine that allows us to understand the IUGR and then the hypotonia at birth and the secondary stunted weight loss. Indeed, hypotonia and/or psychomotor retardation of the infant may have a metabolic cause.

Very recently, Savall et al. in 2015 [14] presented a case of suspicion of methylmalonic aciduria in an infant that allowed us to revisit the diagnosis of neuro-Biermer in his mother in whom there was no hematological sign. However, she had a painful obstetric history (several miscarriages and a baby died of bilateral renal agenesis at birth) and gave birth to a girl of 2.88 kg and 46 cm in January 2015, at 39 WA, healthy outside with slight intrauterine growth retardation [14]. For the patient situation, it is rather the presence of Biermer's disease that allowed the follow-up and the diagnosis of this stunted weight growth retardation. The maternal vitamin B12 deficiency is not always responsible for hematological abnormalities, thus delaying the diagnosis. In our patient, we can discuss the role of hyperhomocysteinemia induced by vitamin B12 deficiency as the cause of the first miscarriage. Barro et al. [15] also reported three similar cases of complex obstetric history and IUGR related to Biermer's disease. Infants born to mothers who are vitamin B12 deficient and exclusively breastfed are at high risk for neurological complications. Neonatal screening for this deficiency through the determination of blood or urinary methylmalonic acid may help avoid these complications and quickly diagnose the maternal cause [14]. We started vitamin B12 supplementation in infants.

## 4. Conclusion

Biermer's disease as a very rare maternal cause of intrauterine growth retardation and infantile hypotonia is becoming a reality in Togo. Vitamin B12 therapy should be provided lifelong to prevent relapses of neurological conditions and fetal consequences that may be irreversible. We advocate special care from birth for children born to mothers with this disease.
